# Basic Competence of Intensive Care Unit Nurses: Cross-Sectional Survey Study

**DOI:** 10.1155/2015/536724

**Published:** 2015-10-18

**Authors:** Riitta-Liisa Lakanmaa, Tarja Suominen, Marita Ritmala-Castrén, Tero Vahlberg, Helena Leino-Kilpi

**Affiliations:** ^1^Turku University of Applied Sciences, 20720 Turku, Finland; ^2^School of Health Sciences, University of Tampere and University of Turku, 33014 Tampere, Finland; ^3^Department of Anesthesiology and Intensive Care, Helsinki University Hospital, HUS, 00029 Helsinki, Finland; ^4^Biostatistics, University of Turku, 20014 Turku, Finland; ^5^Department of Nursing Science, University of Turku, 20014 Turku, Finland; ^6^Turku University Hospital, 20521 Turku, Finland

## Abstract

Critical care patients benefit from the attention of nursing personnel with a high competence level. The aim of the study was to describe and evaluate the self-assessed basic competence of intensive care unit nurses and related factors. A cross-sectional survey design was used. A basic competence scale (Intensive and Critical Care Nursing Competence Scale version 1, Likert scale 1–5, 1 = poor and 5 = excellent) was employed among Finnish intensive care unit nurses (*n* = 431). Intensive care unit nurses' self-assessed basic competence was good (mean 4.19, SD 0.40). The attitude and value base of basic competence was excellent whereas experience base was the poorest compared to the knowledge base and skill base of intensive and critical care nursing. The strongest factor explaining nurses' basic competence was their experience of autonomy in nursing care (*F* value 60.85, *β* 0.11, SE 0.01, and *P* ≤ 0.0001). Clinical competence was self-rated as good. Nurses gave their highest competence self-ratings for ICU patient care according to the principles of nursing care. The ICU nurses also self-rated their professional competence as good. Collaboration was self-rated as the best competence. In basic and continuing education and professional self-development discussions it is meaningful to consider and find solutions for how to improve nurses' experienced autonomy in nursing.

## 1. Introduction

What is known about this topic?

- Competence in intensive and critical care nursing (ICCN) is a multidimensional concept.
- There are, globally, definitions and descriptions of competence in ICCN but only a few studies of intensive care unit nurses' competence.
- Competence evaluation in ICCN education and practice is essential for the nurses' professional development.

What does this paper add?

- Intensive care unit (ICU) nurses' competence contains four bases: knowledge base, skill base, attitude and value base, and experience base. ICU nurses self-evaluated their experience base as the poorest. Experience of ICCN care is crucial for the professional development of the ICU nurses.
- ICU nurses' experience of autonomy in nursing care explains self-evaluated basic competence. Autonomy in nursing care is important for the professional growth of the ICU nurses.
- Comprehensive competence of ICU nurses should in various ways be developed during ICCN education and orientation programs. This should be considered carefully when planning the contents and educational methods for the programs.

There are approximately 30 intensive care units (ICUs) in Finland in which over 28,000 patients are cared for annually [1]. It is estimated that by the year 2030 the need for intensive and critical care will grow by 25%, for example, due to the fact that the Finnish population is getting older [2]. Multifactorial reasons increase the need of critical care beds in Finland and also in other countries in Europe.

Intensive care unit nurses (ICU nurses) are the largest professional group working in the ICUs. Severely ill patients and families benefit from the attention of highly trained and skilled personnel [3, 4]. ICU nurses contribute to patient safety, such as improved patient outcomes, reduced morbidity and mortality, and decreased complications, errors, and overall costs [5–10]. Competent ICU nurses have a significant impact on ICU patient's physiological and psychological outcomes, for example, evidence based nursing interventions and ethical activity.

Critical care nursing is an own nursing speciality [11] whereas technology is integrated with psychosocial challenges and ethical conflicts associated with critical illness [12]. Generally, critical care nursing education is a special postqualification education [13] and is described in the European Qualifications Framework for Lifelong Learning as level 6 (of eight) [14, 15]. Currently no postqualification education in intensive and critical care nursing leading to a degree exists in Finland.

We know that there is a worldwide need for competent nurses [16]. In nursing practice, nurses get to demonstrate on a regular basis clinical competence as well as a sound theoretical knowledge base according to nationally recognized frameworks [4, 17, 18]. There is a need for valid specific instruments (e.g., for intensive and critical care nursing) for competence evaluation purposes. Competence evaluation can be performed in various ways, for example, using self-assessment, knowledge tests, peer evaluation (colleague), observation (mentor, supervisor), OSCE (objective structured clinical examination), or portfolios. The best way to perform competence evaluation is to combine different evaluation methods. Self-assessment is an essential ability that can be learned and developed during education and nursing career.

Intensive and Critical Care Nursing Competence Scale research is scarce. Only three scales (Basic Knowledge Assessment Tool [BKAT], Intensive Care Hundred Item Test [I-HIT], and multilevelled critical care competency statements) of competence in intensive and critical care nursing are reported in nursing literature [19]. Of these scales, two are knowledge tests (BKAT + I-HIT) while the third one is based on nurses' self-assessment; however, we are well aware that it has not been properly tested for reliability and validity and is still in its early phases. The need for a basic competence self-assessment scale in intensive and critical care nursing is therefore also evident, for example, for undergraduate education for beginning registered nurses practice standards. The systematic literature review conducted for this study in PubMed and search from critical care nursing organizations over the last ten years found 21 studies or documents of competence in intensive and critical care nursing (see the appendix).

As a summary of the literature, competence concept is very multidimensional (e.g., focusing on clinical practice [nursing process], ethics, collaboration, leadership, education, and development work) and strongly related to, for example, age, work experience, and frequency of using specific competencies. Competence is studied worldwide (10 documents from Europe, 7 documents from Australia and New Zealand, and 4 documents from USA, Canada, and Brazil); thus, there is lacking systematic competence evaluation research. Evaluation is often performed by self-assessment [20–22] or knowledge test [23]. ICU nurses' self-assessed competence was evaluated as ranging from moderate to excellent: from moderate to good (sample consisting of newly registered ICU nurses) [22], good (sample consisting of ICU nurses with varying work experience) [20], and from good to excellent (sample consisting of ICU nurses with varying work experience) [21]. European ICU nurses' overall knowledge score was 66% of 100 points (min 22, max 91, and SD 12); the main factor that contributed to variance in scores is length of nurses' intensive care experience. The knowledge category with the lowest score was respiration and ventilation [23].

The purpose of this empirical study was to describe and evaluate the self-assessed basic competence of intensive care unit nurses and related factors using a scale, Intensive and Critical Care Nursing Competence Scale version 1 (ICCN-CS-1) [11]. The related factors were divided into the following areas: (i) age and gender, (ii) education, (iii) work experience, (iv) information retrieval, (v) knowledge test, and (vi) others. The hypothesis was that the basic competence can be explained with these. This empirical study is part of a larger project where the scale was developed [19]. This study was conducted to facilitate establishment of the psychometric properties (reliability and construct validity) of the scale being used [24]. The project from which this study is a part aimed to define competence in intensive and critical care nursing (ICCN), to develop a basic self-assessment based measurement scale for competence evaluation in ICCN, and to describe graduating nursing students' basic competence in ICCN by seeking reference basis from ICU nurses.

ICU nurse is a registered nurse who practises in an ICU and whose nurse education is Bachelor of Health Care, specialist nurse, or nurse. In this ICU nurses basic competence study was used also the experience base of the ICU nurses in data analysis which were not analysed in graduating nursing students' basic competence study. The students had no experience base enough in ICCN and therefore in data analysis only three bases of four were analysed knowledge, skill, and attitude and value bases. A novel issue in this study is that it highlights the multidimensionality of the concept of competence. Four bases of competence in intensive and critical care nursing were analysed in ICU nurses' data: knowledge base, skill base, attitude and value base, and experience base [19].

ICCN-CS-1 measures basic competence and it refers to preliminary competence to practice in an ICU. It is comparable to step 1 competencies of the National Competency Framework for Adult Critical Care Nurses [25] and Critical Care Nurse Education Practice Standards [26]. ICCN-CS-1 is useful in planning supervision in preregistration nursing students' clinical practice and during orientation programmes, for example, to identify learning gaps, define learning goals, and discuss competence in a comprehensive manner. The content of the scale is in line with postgraduate education standards and the competence standards of critical care nurses, also at specialist level. The scale measures the basic competence in intensive and critical care nursing and can be used as a starting point for the transition process from preregistration education to postgraduation education. The scale was developed for intensive and critical care education purposes, for example, for preregistration education and in orientation programmes. The results of this study can be useful in nursing education, especially in basic education and when planning continuous education. In clinical practice the results can also be helpful in professional performance appraisals in ICUs.

## 2. Materials and Methods

### 2.1. Aim

The aim of the study was to describe and evaluate the self-assessed basic competence of intensive care unit nurses and related factors. The results of this study will help educators, clinicians, and researchers to develop basic nursing education and orientation in the ICUs.

### 2.2. Research Questions

The research questions for this study were as follows:

1. What is the level of intensive care unit nurses' self-assessed basic competence?
2. What are factors related to the basic competence?

### 2.3. Design

A cross-sectional survey design was employed using questionnaires among Finnish ICU nurses in university hospitals.

### 2.4. Sample/Participants

Total sampling was used for ICU nurses in five Finnish university hospitals (*n* = 431 participants, response rate 54%). In one of the five university hospitals, a convenience sample of ICU nurses (*n* = 82 participants, response rate 37%) also completed a biological and physiological knowledge test. In 2008, a pilot study had been conducted in one university hospital; this organization was therefore not included in this data gathering in 2010. The final number of hospitals taking part in this study was thus four.

### 2.5. Data Collection

Data were collected using a questionnaire with the support of contact persons in the ICUs. The instruments used in this study were the Intensive and Critical Care Nursing Competence Scale (ICCN-CS-1) [24] and a knowledge test, the Basic Knowledge Assessment Tool version 7 (=BKAT-7) [27], which was used as the background factor in the convenience sample. Background factors (12) integral to the ICCN-CS-1 were asked and they formed the first page of the questionnaire.

ICCN-CS-1 is a self-assessment tool consisting of 144 items (six sum variables). The scale measures basic competence in intensive and critical care nursing. Theoretically, basic competence is divided into clinical competence and professional competence. Clinical competence consists of three subdomains: principles of nursing care, clinical guidelines, and nursing interventions. Professional competence consists of four subdomains: ethical activity and familiarity with health care laws, decision-making, development work, and collaboration. In addition, basic competence comprises four bases: knowledge, skill, attitude and value, and experience bases. Each of them has seven subdomains. Every item is measured with the Likert scale 1–5, giving the range score 144–720. Sum of scores on the ICCN-CS-1 can be classified as poor competence (144–288), moderate competence (289–432), good competence (433–576), or excellent competence (577–720) [24]. Mean score can be classified as follows: 1–2.49, poor; 2.5–3.49, moderate; 3.5–4.49, good; or 4.5–5.0, excellent (Table 1) [19].

The 12 background factors for all ICU nurses were divided into five areas: (i) age and gender (2 questions), (ii) education (basic, continuing education, and participation in conferences; 3 questions), (iii) work experience (ICU, other health care; 2 questions), (iv) information retrieval (independent, use of nursing journals; 2 questions), and (v) others (autonomy in nursing and work motivation, on scale 1–10, 1 = poor and 10 = excellent, and special responsibility areas in ICU, scale yes/no). The respondents' experience of autonomy in nursing was asked by requesting them to assess how autonomous they felt their work as a nurse. The convenience sample of ICU nurses (*n* = 82) completed the BKAT-7 as an extra background factor area (vi). BKAT-7 is a biological and physiological knowledge test comprising 100 items. Each correct answer gives one point, yielding the range 0–100. The BKAT has eight sum variables: cardiovascular, monitoring lines, pulmonary, neurology, endocrine, renal, gastrointestinal, and other variables [27]. The data were gathered between January 2010 and May 2010.

### 2.6. Ethical Considerations

The research was conducted according to ethical guidelines [28, 29]. Ethics committee approval was obtained from a university XXX [26.10.2009]. The permission to use BKAT-7 was given by Dr. Jean Toth and to use the Finnish version by MNSc MaritaRitmala-Castrén. Research permission was obtained separately from each participating hospital. Participation was voluntary and based on anonymity in every phase. It was assumed that by returning the questionnaire participants gave their consent to take part in the study. The hospitals were not compared with each other. The data were stored according to ethical guidelines (in safe storage and anonymously).

### 2.7. Data Analysis

The data were statistically analysed using SAS for Windows (version 9.2, SAS Institute Inc., Cary, NC). The sum variables were calculated by dividing the sum score by the number of items answered. To calculate the sum variables and total sum of the ICCN-CS-1 and BKAT-7, 80% of the items should be answered. Two-independent-sample *t*-test was used to test the differences in basic competence between factors that consisted of two categories. The difference in basic competence between education groups was tested with one-way analysis of variance using Tukey's adjustment in pairwise comparisons. Linear associations between continuous background factors and basic competence were analysed using linear regression analysis. Factors associated with basic competence were included in analysis of covariance, except for age which was excluded to avoid multicollinearity problems (age versus work experience, *r* = 0.76), while other work experience was excluded due to the high number of missing values (*n* = 103). Correlations were calculated using the Spearman correlation coefficients. The level of statistical significance was defined as *P* < 0.05.

### 2.8. Validity and Reliability

Reliability and validity of the ICCN-CS-1 have been tested and were shown to be adequate for the scale at its early stage. The scale is based on an extensive theoretical phase where the content of competence was defined [24]. In this study the internal consistency of ICCN-CS-1 was 0.99 (range of sum variables 0.88–0.98), which showed adequate reliability. The reliability and validity of the scale need further developing and testing. As the BKAT is a knowledge test, its internal consistency was not evaluated.

## 3. Results

### 3.1. Participants

Most participants were female (84.6%) and their mean age was 38 years (range 22–62). Most of the nurses had a Bachelor of Health Care degree (52.9%) and their mean length of work experience in ICU was 9.1 years (0.02–36 years) (Table 2).

### 3.2. ICU Nurses' Basic Competence

The nurses' (*n* = 431) self-rated basic competence was 67.5% excellent, 32.3% good, and 0.2% moderate. The mean score was 4.19 (2.79–5.00, SD 0.40). Clinical competence (directly related to patient care) was self-rated as good. Nurses gave their highest competence self-ratings for ICU patient care according to the principles of nursing care (this refers to ethical standards such as safety, justness, patient centeredness, recognition of abnormal vital signs, need of pain care, changes in skin condition, and need of fluid therapy). The ICU nurses self-rated their professional competence (related to the profession in general) as good. Collaboration was self-rated as the best competence and development work as the poorest. Clinical competence was rated as better than professional competence. The nurses self-rated their knowledge base, skill base, and experience base as good. Attitude and value base was rated as excellent. The nurses gave the poorest ratings for the experience base of competence (Table 3).

### 3.3. Background Factors in relation to Basic Competence

Of the twelve background factors ten were positively associated with basic competence (Table 4). The values obtained from univariable models, ANCOVAs, when forming ANCOVA model (*n* = 405) where age and other work experience were excluded (age and work experience were highly correlated, *r* = 0.76, and other work experience had 103 missing values), autonomy in nursing care (*F* value 60.85, *β* 0.11, SE 0.01, *P* ≤ 0.0001), special responsibility areas in the ICU (*F* value 9.33, *β* 0.13, SE 0.04, and *P* = 0.0024), work experience in ICU (*F* value 7.73, *β* 0.008, SE 0.003, and *P* = 0.0057), independent information retrieval of intensive and critical care nursing (*F* value 7.07, *β* 0.17, SE 0.07, and *P* = 0.0082), and participation in intensive care conferences and education days (*F* value 5.78, *β* 0.10, SE 0.04, and *P* = 0.0167) remained significant. More education (basic and continuing education) and information retrieval (use of nursing journals) were positively related to basic competence, whereas work motivation or gender had a negative association with it.

A knowledge test, BKAT-7, was used as the background factor in this study in a convenience sample of ICU nurses. The ICU nurses' mean score of BKAT-7 was (*n* = 82) 68.26 (SD 10.27, range 32–86) and the ICCN-CS-1 mean score was 4.13 (SD 0.42, range 3.02–5.00). No correlation was detected between knowledge test and self-assessment basic competence scale (*r* = 0.098).

## 4. Discussion

This study used a self-assessment based competence scale which is developed directly for intensive and critical care nursing. It is clinically important that nursing competence in specific areas of nursing is evaluated with the help of specific competence scales.

ICU nurses' self-assessed basic competence was in general good and was related to work experience as found in previous studies [20–22]. They rated their clinical competence higher than professional competence. The reason for this may be the high technical and clinical skill requirements in the ICUs. Also ethics was highlighted in many ways: ICU nurses self-assessed their attitude and value base as excellent. In clinical competence, “principles of nursing care” was rated as high while in professional competence “ethical activity and familiarity with health care laws” was rated as low. This can be explained by the fact that ICU nurses at bed-side nursing care are more familiar with safe, direct patient-centred care (when using in practice these competencies frequently in daily care) than with adherence to ethical codes, general health care legislation, and transplantation legislation (literally) [20–22].

In professional competence, collaboration was self-assessed as the best and development work (referring to self-development, team development, nursing development, and subordinate skill development) as the poorest dimension of competence. This finding shares similarities with several previous studies [20–22]. One reason may be that nurses lack education, experience, and resources for development work in their profession. In addition, nurses' attitudes towards evidence based nursing and development work may be negative because they do not see themselves as “developers,” merely as “clinical workers.” They might also think that these kinds of jobs are not for them, or they may not have enough knowledge of them.

In experience base the nurses were guided to evaluate how sufficient they assess the quality of their experience in specific issues in intensive and critical care nursing (“Do the nurses have enough experience in every aspect of competence?”) The nurses regarded this with suspicion. They evaluated the quality and sufficiency of their experience base very critically. The researchers interpreted this as denoting that special competence in nursing is associated with extensive experience. The autonomy in nursing experienced by nurses was the strongest factor related to basic competence. This finding is logical and natural because autonomy is an essential element of professional status as stated by Varjus et al. in their review [30].

The basic biological and physiological knowledge did not correlate with self-assessed basic competence. In this study the ICU nurses' mean of BKAT-7 knowledge test was 68 of 100 (SD 10), which correlates to a previous study by Fulbrook et al. [23] (Finnish nurses' mean score was 64 of 100, SD 9; in this European ICU nurses study, the I-HIT knowledge test was used). Competence as a concept is holistic and multidimensional, with knowledge only a part of the whole. This gives an idea for competence evaluation and planning in nursing education. “Knowledge base comes first,” but also other fields of competence such as skill base, attitude and value base, and experience base as well as personal base of the ICU nurse are important [11].

Most of the ICU nurses search for information independently and participate in conferences and education days. The variance in work motivation and autonomy in nursing was 2–10, which means that some nurses have very low motivation and do not feel autonomy in their work. In this study, work motivation was not associated with self-assessed basic competence whereas autonomy was strongly associated with it. It is important that the relationship between “safety culture” and “basic competence” is examined together and attended to in ICUs. Nurses' competence is related to intent to leave nursing profession, job satisfaction, patient safety, and quality of nursing in ICUs [31].

### 4.1. Strengths and Limitations

There are limitations in this study. They are related to the national sample, sampling which covered only university hospitals and the somewhat low response rate. The basic competence scale ICCN-CS-1 was a new scale and it shares the same difficulties with a previous holistic competence scale developed for critical care nursing [32]. The Cronbach alpha values were somewhat high and the nature of the competence scale is unidimensional, and the items correlate strongly with each other [19]. Furthermore, the method of self-assessment is questionable: how reliable is self-assessment as a method? Self-assessment as a skill is important for nurses; however, it should not be used alone but in combination with other assessment methods. It is also important to recognize the bias related to respondents. Dropout analysis was not performed. However, the scale was developed very carefully by experts in an academic setting and the sampling was planned to cover the most competence-demanding ICUs in Finland. The response rate was also quite good for a questionnaire study.

## 5. Conclusions

Firstly, ICU nurses' experienced autonomy in nursing care is highly related to self-assessed basic competence. Secondly, specific responsibility areas in ICUs (such as medical emergency team member, student supervisor) and, thirdly, work experience in the ICU are related to self-assessed basic competence. In basic and continuing education and professional self-development discussions it is meaningful to consider and find solutions for how to improve nurses' experienced autonomy in nursing and how to find specific interest areas for every ICU nurse and motivate as well as retain them in intensive and critical care nursing. Fourthly, work experience in years as well as the quality of work experience (sufficiency) is an essential part of basic competence in intensive and critical care nursing. Fifthly, there are domains in ICU nurses' basic competence that score lower than other domains and bases, such as “experience base of competence,” “development work,” and “ethical activity and familiarity with health care laws,” which can be highlighted in education planning.

## Figures and Tables

**Table 1:** Structure and item amounts of ICCN-CS-1.

Basic competence = clinical competence + professional competence	Knowledge base	Skill base	Attitude and value base	Experience base	*Items*
Clinical competence (80)					
Principles of nursing care	4	4	4	4	16
Clinical guidelines	4	4	4	4	16
Nursing interventions	12	12	12	12	48
Professional competence (64)					
Ethical activity and familiarity with health care laws	4	4	4	4	16
Decision-making	4	4	4	4	16
Development work	4	4	4	4	16
Collaboration	4	4	4	4	16
* Items*	36	36	36	36	144

**Table 2:** Characteristics of nurses (*n* = 431).

Background factors	Nurses			
	Mean	SD	Min	Max
	*n*		%	
Age (*n* = 430)	38	9.9	22	62
Gender (*n* = 421)				
Female/male	356/65		84.6/15.4	
Education (*n* = 429)				
Nurse (Bachelor of Health Care)	227		52.9	
Specialist nurse	95		22.1	
Nurse	82		19.1	
Others	25		5.8	
Work experience (years) as a nurse in intensive and critical care (*n* = 425)	9.1	8.1	0.02	36
Other work experience as a nurse in health care (*n* = 328)	5.4	7.2	0	37
Continuing education in intensive care nursing (*n* = 423)				
Yes	73		17.3	
No	350		82.7	
Participation in intensive care conferences and education days (*n* = 428)				
Yes	307		71.7	
No	121		28.3	
Independent information retrieval of intensive and critical care nursing (*n* = 426)				
Yes	400		93.9	
No	26		6.1	
Use of nursing journals in information retrieval of intensive and critical care nursing (*n* = 429)				
Yes	367		86.0	
(i) International scientific journals	67		18.3	
(ii) National scientific journals	141		38.4	
(iii) Professional journals	352		95.9	
No	62		14.0	
Work motivation (1–10) (*n* = 429)	8.1	1.5	2	10
Autonomy in nursing (1–10) (*n* = 430)	8.1	1.2	2	10
Special responsibility areas in the ICU (*n* = 428)				
Yes	326		76.2	
No	102		23.8	

**Table 3:** The domains and bases of basic competence and the self-assessment scores.

Domains and bases of basic competence	Self-assessment scores (1–5)	
	(*n* = 428–431)	
	Mean	SD
Basic competence	4.19	0.40
Clinical competence	4.33	0.39
Principles of nursing care	4.47	0.41
Clinical guidelines	4.36	0.38
Nursing interventions	4.27	0.44
Professional competence	4.02	0.45
Collaboration	4.28	0.45
Decision-making	4.24	0.48
Ethical activity and familiarity with health care laws	3.90	0.55
Development work	3.65	0.59
Attitude and value base of competence	4.68	0.32
Knowledge base of competence	4.05	0.45
Skill base of competence	4.02	0.46
Experience base of competence	3.82	0.68

**Table 4:** Statistically significant background factors in relation to basic competence.

Background factor	*P* value	Regression coefficient *β*	Standard error	Mean of ICCN-CS-1	SD
Age (*n* = 430)	<0.0001^1^	0.02	0.002		
Education (*n* = 429)	<0.0001^2^				
Nurse (Bachelor of Health Care)				4.07	0.38
Specialist nurse				4.39	0.32
Nurse				4.35	0.36
Others				4.04	0.43
Work experience (years) as a nurse in intensive and critical care (*n* = 425)	<0.0001^3^	0.02	0.002		
Other work experience as a nurse in health care (*n* = 328)	0.0004^1^	0.01	0.003		
Continuing education in intensive care nursing (*n* = 423)	<0.0001^3^				
Yes				4.35	0.40
No				4.15	0.39
Participation in intensive care conferences and education days (*n* = 428)	<0.0001^3^				
Yes				4.30	0.35
No				3.92	0.39
Independent information retrieval of intensive and critical care nursing (*n* = 426)	0.0001^3^				
Yes				4.21	0.39
No				3.90	0.43
Use of nursing journals (*n* = 429)	<0.0001^3^				
Yes				4.23	0.38
No				4.00	0.43
Autonomy in nursing care (1–10) (*n* = 429)	<0.0001^1^	0.16	0.01		
Special responsibility areas in the ICU (*n* = 428)	<0.0001^3^				
Yes				4.28	0.36
No				3.90	0.39

^1^Linear regression.

^2^One-way analysis of variance; Tukey's adjusted *P* values; nurse (BHC) versus specialist nurse (*P* < 0.0001); nurse (BHC) versus nurse (*P* < 0.0001); specialist nurse versus others (*P* = 0.0001); nurse versus others (*P* = 0.001).

^3^Two-independent-sample *t*-test.

**Table 5:** Summary of competence literature in intensive and critical care nursing 2004–2014.

Organisation/author, year, country	Document/title of the study/method	Aim	Main findings/results
Gill et al. 2015, Australia [26]	“Development of Australian Clinical Practice Outcome Standards for Graduates of Critical Care Nurse Education” Delphi technique	To develop critical care nurse education practice standards	The process resulted in the development of 98 practice standards, categorized into three levels

Lakanmaa et al. 2014, Finland [24]	“Basic Competence in Intensive and Critical Care Nursing: Development and Psychometric Testing of a Competence Scale” Questionnaire survey	To develop a scale to assess basic competence in intensive and critical care nursing	The Intensive and Critical Care Nursing Competence Scale is a self-assessment test consisting of 144 items. Basic competence is divided into patient-related clinical competence and general professional competence. Basic competence consists of knowledge base, skill base, attitude and value base, and experience base

EfCCNa 2013, Europe [4]	“EfCCNa Competencies for European Critical Care Nurses”	To develop a European Critical Care Nursing competency framework	Four main domains: clinical domain, professional domain, managerial domain, and education and development domain. These are divided into 14 different subdomains

Camelo 2012, Brazil [33]	“Professional Competences of Nurse to Work in Intensive Care Units: An Integrative Review” Literature review	To identify and analyse nurses' competences to work at intensive care units	Eight themes of competence were found: nursing care management, high-complexity nursing care delivery, decision-making, leadership, communication, continuing/permanent education, human resource management, and material resource management

Gill et al. 2012, Australia [34]	“A Review of Critical Care Nursing Staffing Education and Practice Standards” Review	To review the differences and similarities in critical care nursing staffing, education, and practice standards in the US, Canada, UK, New Zealand, and Australia	There is a general consensus about the importance of optimum staffing by registered nurses with proportion of those holding relevant postregistration qualifications; there is no consistency in defining the educational preparation for qualified critical care nurse

Hadjibalassi et al. 2012, Cyprus [35]	“Development of an Instrument to Determine Competencies of Postgraduate ICU Nurses in Cyprus Combination of Qualitative and Quantitative Approach”	To report the development of an instrument to determine what competencies are expected of postgraduate critical care nurses	The final questionnaire includes 72 items and has a four-dimensional structure; the dimensions are (i) leadership/management and professional development, (ii) decision-making and management of emergencies, (iii) provision of care and professional practice, and (iv) ethical practice

Fullbrook et al. 2012, Australia [23]	“A Survey of European Intensive Care Nurses' Knowledge Levels” Questionnaire survey	To examine the knowledge levels of European intensive care nurses	The overall mean knowledge score was 66% (SD 12); the main factor that contributed to variance in scores was nurses' length of intensive care experience; the knowledge category which scored lowest was respiration and ventilation

Lakanmaa et al. 2012, Finland [11]	“Competence Requirements in Intensive and Critical Care Nursing-Still in Need of Definition? A Delphi Study” Qualitative Delphi study	To identify competence requirements	Competence requirements can be divided into five main domains: knowledge base, skill base, attitude and value base, nursing experience base, and personal base of the nurse

Critical Care Networks-National Nurse Leads 2012, UK [25]	“National Competency Framework for Adult Critical Care Nurses”	The framework is a collection of the core clinical competencies that have been identified as basic to the effective performance of adult critical care nursing	Step 1 competencies should be commenced when a nurse begins in critical care or when he/she has no previous experience of the speciality Step 2 and 3 competencies should be completed during the period of an academic critical care programme The Critical Care Competency Framework Content includes several system and additional areas

O'Leary 2012, USA [21]	“Comparison of Self-Assessed Competence and Experience among Critical Care Nurses” Questionnaire survey	To determine the level of self-assessed nursing competence and the relationship with age and experience in nursing	The nurses “self-assessed level of competence ranged from good to excellent along with an increased frequency of using competencies. The longer the nurses” experience, the greater their self-assessed level of competence

Stewart and Rae 2013, UK [36]	“Critical Care Nurses' Understanding of the NHS Knowledge and Skills Framework. An Interpretative Phenomenological Analysis” Qualitative study	To explore critical care nurses' understanding of the National Health Service (NHS) Knowledge and Skills Framework (KSF)	Two superordinate themes of “engagement” and “theory-practice gap” were identified; six subthemes of “fluency,” “transparency,” “self-assessment,” “achieving for whom,” “reflection,” and “the nursing role” further explained the superordinate themes Challenges identified were primarily concerned with complex language, an unclear process, and the use of reflective and self-assessment skills

Critical Care Nurses' Section 2010, New Zealand [37]	“New Zealand Standards in Critical Care Nursing Education”	The standards provide the framework for curriculum development and student evaluation	There are six standards: (i) nursing education is provided and managed by appropriately qualified staff, (ii) entry requirements for nursing programmes are explicit, fair, and equitable, (iii) the curriculum is developed collaboratively and directed towards providing clinical, educational, and professional preparation to be a qualified nurse, (iv) the opportunity to gain clinical competence in the areas covered by the programme is also provided, (v) nurses are assessed throughout and on completion of the programme, and (vi) theoretical content is offered to provide the nurse with knowledge to assess, plan, manage, document, and analyse the care of the critically ill patient and family

CACCN 2009, Canada [38]	“Standards for Critical Care Nursing Practice”	To provide an essential resource to all nursing professionals in their pursuit of best practice in the critical care environment	Seven standards are provided related to patient monitoring and management for the promotion of optimal physiological balance, comfort, and well-being of the patient, patient and family centeredness care, end-of-life care, patient safety and best practice, collaboration practice, and leadership

AACN 2008, USA [39]	“AACN Scope and Standards for Acute and Critical Care Nursing Practice”	To describe a competent level of behaviour in the professional role. The measurement criteria describe how the standards are met	The nursing process is used as the framework. Nine standards include activities related to quality of professional practice, professional practice evaluation, education, collegiality, ethics, collaboration, research, resource utilization, and leadership

Ääri et al. 2008, Finland [40]	“Competence in Intensive and Critical Care Nursing: A Literature Review” Literature review	To define and describe the concept of competence in adult intensive care nursing	Clinical and professional competence in intensive and critical care nursing can be defined as a specific knowledge base, skill base, attitude and value base, and experience base of intensive and critical care nursing

Salonen et al. 2007, Finland [22]	“Competence Profiles of Recently Registered Nurses Working in Intensive and Emergency Settings”	To describe recently registered nurses' perceptions of their competence level and to identify factors influencing these perceptions	Nurses' self-assessed competence level ranged from moderate to good; a statistically significant association was seen between competence level and age, length of current work experience, and the frequency of using competencies

ACCCN 2006, Australia [41]	“ACCCN Position Statement (2006) on the Provision of Critical Care Nursing Education”	To outline the recommendations regarding the provision of critical care nursing education	The recommendations are based on evidence from research in critical care nursing or allied fields In areas where current research-based evidence is not available, the recommendations (16, 16 subject areas) are based on the opinion of expert nurses

Lindberg 2006, Sweden [42]	“Competence in Critical Care: What It Is and How to Gain It: A Qualitative Study from the Staff's Point of View” Qualitative interview study	To contribute to the body of knowledge relating to the concept of competence	Five different ways of understanding competence in intensive care were described: ability to cooperate, being able to perceive the situation correctly, being aware of abilities and limitations, being able to act, and being able to disregard the technology when needed

Fisher et al. 2005, Australia [32]	“Competency Standards for Critical Care Nurses: Do They Measure Up?” Questionnaire survey	To determine the construct validity of the Australian College of Critical Care Nurses (ACCCN) competency standards as a tool for assessing the clinical practice of specialist level critical care nurses	There was no support for the structure for the ACCCN competencies; the elements did not fit statistically uniquely to a single competency. Competency statements also loaded across several domains

WFCCN 2005 [43]	“Position Statement on the Provision of Critical Care Nursing Education-Declaration of Madrid, 2005”	To inform critical care nursing associations, health care providers, and educational facilities of the development and provision of critical care nursing education	Five central principles and 14 recommendation guidelines providing critical care nursing education: health services, educational facilities, and critical care nursing organisations

Meretoja et al. 2004, Finland [20]	“Comparison of Nurse Competence in Different Hospital Work Environments” Questionnaire survey	To examine nurses' perceptions of competence in different university hospital work environments	Nurses reported their overall level of competence as good; they felt most competent in the categories of managing situations, diagnostic functions, and helping role and least competent in ensuring quality category The greater the self-assessed level of competence, the higher the frequency of using of competencies; correlations between both age and length of work experience and the self-assessed overall level of competence were positive

## Appendix

See Table 5.

## References

[1] Ritmala-Castrén M., Lundgren-Laine H., Murtola L.-M. (2014). Aikuispotilaiden tehohoitopalvelut Suomessa vuonna 2012. *Tehohoito*.

[2] Reinikainen M. (2012). *Hospital mortality of intensive care patients in Finland. Insights into prognostic factors and measuring outcomes [Ph.D. thesis]*.

[3] EfCCNa Position Statement on workforce requirements within. http://www.efccna.org/downloads/Position%20Statement%20Workforce%20EfCCNa%202007.pdf.

[4] EfCCNa (2013). *EfCCNa Competencies for European Critical Care Nurses*.

[5] Robnett M. K. (2006). Critical care nursing: workforce issues and potential solutions. *Critical Care Medicine*.

[6] West E., Mays N., Rafferty A. M., Rowan K., Sanderson C. (2009). Nursing resources and patient outcomes in intensive care: a systematic review of the literature. *International Journal of Nursing Studies*.

[7] Penoyer D. A. (2010). Nurse staffing and patient outcomes in critical care: a concise review. *Critical Care Medicine*.

[8] Kendall-Gallagher D., Blegen M. A. (2009). Competence and certification of registered nurses and safety of patients in intensive care units. *American Journal of Critical Care*.

[9] Person S. D., Allison J. J., Kiefe C. I. (2004). Nurse staffing and mortality for medicare patients with acute myocardial infarction. *Medical Care*.

[10] Rischbieth A. (2006). Matching nurse skill with patient acuity in the intensive care units: a risk management mandate. *Journal of Nursing Management*.

[11] Lakanmaa R.-L., Suominen T., Perttilä J., Puukka P., Leino-Kilpi H. (2012). Competence requirements in intensive and critical care nursing—still in need of definition? A Delphi study. *Intensive and Critical Care Nursing*.

[12] Relf M., Kaplow R., Gonce M. P., Fontaine D. K., Hudak C. M., Gallo B. M. (2006). Critical care nursing practice: an integration of caring, competence, and commitment to excellence. *Critical Care Nursing. A Holistic Approach*.

[13] WHO (2003). *WHO Europe Critical Care Nursing Curriculum*.

[14] Ministry of Education (2009). *The National Framework for Qualifications and Other Learning*.

[15] European Communities https://ec.europa.eu/ploteus/sites/eac-eqf/files/leaflet_en.pdf.

[16] Flinkman M. (2014). *Young registered nurses intent to leave the profession in Finland—a mixed method study*.

[17] ACCCN (2006). *Position Statement (2006) on the Provision of Critical Care Nursing Education*.

[18] EfCCNa Position Statement on Post-registration Critical Care Nursing Education within Europe. http://www.efccna.org/downloads/Position%20statement%20on%20education%20EfCCNa.pdf.

[19] Lakanmaa R.-L. (2012). *Competence in intensive and critical care nursing—development of basic assessment scale for graduating nursing students [dissertation]*.

[20] Meretoja R., Leino-Kilpi H., Kaira A.-M. (2004). Comparison of nurse competence in different hospital work environments. *Journal of Nursing Management*.

[21] O'Leary J. (2012). Comparison of self-assessed competence and experience among critical care nurses. *Journal of Nursing Management*.

[22] Salonen A. H., Kaunonen M., Meretoja R., Tarkka M.-T. (2007). Competence profiles of recently registered nurses working in intensive and emergency settings. *Journal of Nursing Management*.

[23] Fulbrook P., Albarran J. W., Baktoft B., Sidebottom B. (2012). A survey of European intensive care nurses' knowledge levels. *International Journal of Nursing Studies*.

[24] Lakanmaa R.-L., Suominen T., Perttilä J., Ritmala-Castrén M., Vahlberg T., Leino-Kilpi H. (2014). Basic competence in intensive and critical care nursing: development and psychometric testing of a competence scale. *Journal of Clinical Nursing*.

[25] Critical Care Networks-National Nurse Leads National Competency Framework for Adult Critical Care Nurses. http://www.cc3n.org.uk/competency-framework/4577977310.

[26] Gill F. J., Leslie G. D., Grech C., Boldy D., Latour J. M. (2015). Development of Australian clinical practice outcome standards for graduates of critical care nurse education. *Journal of Clinical Nursing*.

[27] Toth J. (2014). *The Basic Knowledge Assessment Tool (BKAT)*.

[28] European Comission (2013). *Ethics for Researchers. Facilitating Research Excellence in FP7*.

[29] TENK (2012). *Hyvä tieteellinen käytäntö ja sen loukkausepäilyjen käsitteleminen Suomessa*.

[30] Varjus S.-L., Leino-Kilpi H., Suominen T. (2011). Professional autonomy of nurses in hospital settings—a review of the literature. *Scandinavian Journal of Caring Sciences*.

[31] Chaboyer W., Chamberlain D., Hewson-Conroy K. (2013). Safety culture in Australian intensive care units: establishing a baseline for quality improvement. *American Journal of Critical Care*.

[32] Fisher M. J., Marshall A. P., Kendrick T. S. (2005). Competency standards for critical care nurses: do they measure up?. *Australian Journal of Advanced Nursing*.

[33] Camelo S. H. H. (2012). Professional competences of nurse to work in intensive care units: an integrative review. *Revista Latino-Americana de Enfermagem*.

[34] Gill F. J., Leslie G. D., Grech C., Latour J. M. (2012). A review of critical care nursing staffing, education and practice standards. *Australian Critical Care*.

[35] Hadjibalassi M., Papastavrou E., Lambrinou E. (2012). Development of an instrument to determine competencies of postgraduate ICU nurses in Cyprus. *Nursing in Critical Care*.

[36] Stewart L. F. M., Rae A. M. (2013). Critical care nurses' understanding of the NHS knowledge and skills framework. An interpretative phenomenological analysis. *Nursing in Critical Care*.

[37] Critical Care Nurses' Section (2010). *New Zealand Standards in Critical Care Nursing Education*.

[38] CACCN (2009). *Standards for Critical Care Nursing Practice*.

[39] AACN (2008). *AACN Scope and Standards for Acute and Critical Care Nursing Practice*.

[40] Ääri R.-L., Tarja S., Helena L.-K. (2008). Competence in intensive and critical care nursing: a literature review. *Intensive and Critical Care Nursing*.

[41] ACCCN (2006). *ACCCN Position Statement (2006) on the Provision of Critical Care Nursing Education*.

[42] Lindberg E. (2006). Competence in critical care: what it is and how to gain it: a qualitative study from the staff's point of view. *Dimensions of Critical Care Nursing*.

[43] WFCCN (2005). *Position Statement on the Provision of Critical Care Nursing Education—Declaration of Madrid, 2005*.

